# Penetration and ligament formation of viscoelastic droplets impacting on the superhydrophobic mesh

**DOI:** 10.1038/s41598-022-15645-1

**Published:** 2022-07-13

**Authors:** Abbasali Abouei Mehrizi, Shiji Lin, Lijie Sun, Yile Wang, Longquan Chen

**Affiliations:** School of Physics, University of Electronic Science and Technology of China, Chengdu, 610054 China

**Keywords:** Chemical engineering, Fluid dynamics

## Abstract

Spraying occurs by the impact of water droplets on the superhydrophobic wire meshes by liquid penetration during the spreading and recoiling. We have shown that adding a small amount of high molecular weight polymer (PEO) alters the ligaments formation and stabilizes them due to its high elasticity. Consequently, it suppresses droplet spray during droplet spreading and recoiling (recoil penetration). In the wide range of the impact velocities, the penetrated ligaments retracted back to the mesh after reaching the maximum length and eventually merged with the droplet on the mesh. The empirical fitting shows that the ligament evolution follows the parallel spring-dashpot model of Kelvin–Voigt. The additive polymer also changes the recoil penetration mechanisms from cavity collapse to cavity detachment due to the higher retraction velocity of the cavity near the mesh that is induced by the upward flow formed by the retraction of the ligaments to the mother droplet. A model based on mass conservation is proposed to calculate the variation of the maximum ligament size.

## Introduction

The impact of liquid droplets on solid surfaces has received growing attention since the pioneering work by Worthington^1^, due to its important and widespread applications in many technological processes, ranging from rainfall-induced erosion^2^ and additive inkjet printing^3^ to surface charge printing^4^ and electricity generation^5,6^. Existing studies have revealed that the dynamics of an impinging droplet is not only influenced by its physical properties but also affected by the surface wettability^7,8^, the substrate stiffness^9^, and the ambient atmospheric pressure^10,11^. In particular, the characteristics of the impact processes and outcomes can be effectively altered by tuning the wetting property of solid surfaces^12,13^. Apart from the droplet deposition at low impact velocities and splash at high impact velocities, which are common for hydrophilic and hydrophobic surfaces^14,15^, novel physical phenomena such as complete rebound, partial rebound, and receding breakup have been successively identified on superhydrophobic surfaces with increasing impact velocity^16,17^. Additionally, within a certain range of impact velocity, an impinging droplet of a low-viscosity liquid on the superhydrophobic surface would be accompanied by the emission of a singular jet and the entrapment of a sub-millimeter-sized bubble^18–20^.

Numerous recent studies have shown that the topography of solid surfaces also plays a non-ignorable role in determining the dynamic behaviors of impinging droplets, and this effect is rather noticeable if the solid substrates are leaky^12^. Lorenceau and Quéré investigated the impact of a liquid droplet on a thin plate with a single hole, and found that the liquid would penetrate the hole to form a long ligament during droplet spreading when the hydrodynamic impact pressure exceeds the capillary pressure^21^. Brunet et al. ^22^ impacted water droplets on hydrophobic microgrids of micron-sized holes and showed that the impact-induced water ligaments could destabilize into tiny droplets with sizes similar to the individual hole size, indicating a potential approach to produce monodisperse sprays. The formation of spray droplets was also reported during the spreading of impinging droplets on other hydrophilic and hydrophobic metal meshes or textiles^23,24^; yet Kooij and co-authors recently demonstrated that the size distribution of the produced spray droplets is not monodisperse, but spans over a broad range^25^. By impinging water droplets on superhydrophobic meshes, Ryu et al.^26^ first identified the generation of liquid ligaments and sprays during droplet recoiling. This novel phenomenon was subsequently confirmed by other experimental works^27,28^. Among them, Sun et al.^28^ suggested that the water spray produced during droplet recoiling is formed by the collapse of an air cavity, which is generated via the development, propagation, and oscillation of the capillary wave upon droplet impact. Moreover, they found that the size distribution of the sprayed droplets depends on the impact velocity and the pore size.

In these works mentioned above, research efforts have been chiefly devoted to studying the dynamic behaviors of impinging Newtonian droplets on porous meshes, particularly water droplets on superhydrophobic meshes. By contrast, the impact of non-Newtonian droplets such as viscoelastic droplets, which can be encountered in practical applications, has received less attention. For example, a certain amount of flexible polymers is always added to enhance the viscoelasticity and retention of dispensing droplets in agricultural spray^29^ and spray cooling^30^.

In a recent study published by our group^31^, the viscoelastic droplet impact on the superhydrophobic mesh has been investigated experimentally. We have tried to introduce and categorize all the impact phenomena, including deposition, rebound, bubble formation without droplet penetration, bubble formation with droplet penetration, penetration, and detachment. The phase diagram of the impact was presented to show the occurrence threshold of each phenomenon. The ligament formation and the sources of their destabilization to the crest swell droplets were studied. Two main perturbation sources, namely the vibration induced by droplet impact on the mesh and the perturbation induced by the cavity collapse, were reported to be responsible for the ligament destabilization. The maximum spreading dynamics were monitored and modeled.

Following the previous study, we investigated the viscoelastic droplet impact on superhydrophobic mesh with different pore sizes S = 357 μm, 135 μm, and 78 μm (Fig. S1). The present study mainly focused on the ligament formation during the spreading and recoiling and the underlying mechanism of the recoil penetration of PEO droplets and compared it with water droplets. We demonstrate that a tiny amount of polymer additives does not affect the onset and occurrence of liquid penetration during droplet spreading, but it suppresses the spray formation by stabilizing the ligaments with elastic forces. The produced ligaments eventually retract back into the mother droplet instead of fragmentation. An empirical model for the ligament evolution and the maximum ligament size have been proposed. Polymer additives also limit the recoil penetration and ultimately suppress it. The underlying mechanism can be explained by changing the dynamics of the air cavity during the retraction, which is different from the cavity collapse mechanism in water droplets. Spray and splash suppression have many applications when the droplet deposition is favorable, and spray/splash should be avoided due to hygiene or protection considerations such as pesticide application and toxic fluids or the Covid 19 transmission by microdroplets^32–34^. In the case of polymer additive into a water droplet, as presented in a recent study, it can be used to suppress the microdroplets generated by using the ultrasonic scaler and dental handpiece during dental filling^34^.

## Results

### Penetration during the spreading

**Figure 1:**
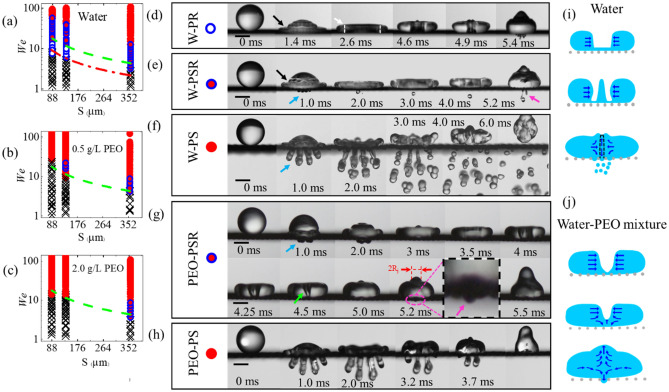
Phase diagram of the liquid penetration (**a**) water, (**b**) 0.5 g/L, and (**c**) 2.0 g/L PEO aqueous solution into the superhydrophobic mesh plotted in terms of the Weber number and the mesh pore wide S. The symbols represent different penetration phenomena which are defined in (**d–h**), symbol (x) shows no penetration. The green dashed line plots the threshold Weber number (*We*_*cr1*_) for liquid penetration during droplet impact (${P}_{D}={P}_{c}$), while the red dashed-dotted line plots the experimental threshold of Weber number (*We*_*cr2*_) for water droplet recoil penetration. (**d–h**) Time sequence photos of different types of penetration for (**d–f**) water droplets and (**d–h**) the PEO aqueous droplets impact on the superhydrophobic mesh when in (**d**) water penetrates the mesh during droplet recoiling (W-PR) at *V*_*0*_ = 0.9 m/s; (**e**) water penetrates the mesh during both droplet spreading and recoiling (W-PSR) at *V*_*0*_ = 1.15 m/s, (**f**) water penetrated the mesh during droplet spreading (W-PS) at *V*_*0*_ = 1.3 m/s, (**g**) PEO aqueous droplet penetrates the mesh during both spreading and recoiling (PEO-PSR) at *V*_*0*_ = 0.58 m/s and PEO concentration 0.5 g/L; (**h**) PEO aqueous droplet penetrates the mesh only during spreading (PEO-PS) at *V*_*0*_ = 1.39 m/s and PEO concentration 3 g/L, the ligament retract back to their mother droplets. The penetration during the spreading and recoiling are denoted by blue and pink arrows, respectively. The scale bars in (**d–h**) are 1.0 mm. (**i**) Schematic diagram of the symmetry cavity collapse for water droplet observed at 4.6–5.2 ms in (**d**). (**j**) Schematic diagram of the cavity detachment observed for PEO aqueous solution at 4.0–5.25 ms in (**g**).

It is well known that an impinging droplet would penetrate the mesh pores upon impact when its velocity is sufficiently high^23,25,26,35^. This liquid penetration proceeds with droplet spreading and has been identified for pure water and diverse aqueous PEO solution droplets in our experiments (Video S1, S2). The average threshold Weber number ${We}_{c1}$, above which liquid penetration occurs, was found to decrease from 15.3 on the superhydrophobic mesh with 87 µm to 3.5 on the superhydrophobic mesh with 357 µm, and does not show notable changes with the addition of PEO additives, as illustrated in Fig. 1a–c and Fig. S2. The nonlinear dependence of ${We}_{c1}$ on pore size can be described by balancing the hydrodynamic pressure induced by droplet inertia (${P}_{D}\sim \rho V_{0}^{2}$) with the capillary pressure (${P}_{C}\sim \frac{\gamma 4 {\text{S}}}{A} \text{cos}{\theta }_{a}$), yielding ${We}_{\text{c1}}\sim \frac{4{D}_{0}}{\text {S}}{\cos}{\theta }_{a}$. Here $\rho$ is the liquid density, $\gamma$ is the surface tension, S is the mesh pore size, $A\sim {\text {S}}^{2}$ is the area of the mesh pore,*V*_0_ is impact velocity, *D*_0_ is the droplet diameter before impact and ${\theta }_{a}$ is the advancing contact angle on the flat superhydrophobic surface. As comparatively shown in Fig. 1a–c and Fig. S2, a good agreement between the modeling prediction and experimental data is obtained.

### Penetration during the recoiling

Similar to previous studies^26^, the occurrence of liquid penetration during droplet recoiling was also observed for pure water on all mesh surfaces (see Video S3, S4), and the corresponding threshold Weber number ${We}_{C2}$ decreases from 7.5 for S = 87 µm to 3.2 for S = 357 µm, which is apparently lower than ${We}_{c1}$ on any given mesh surface (see Fig. 1a). By contrast, we only identified such liquid penetration for aqueous PEO solution droplets (referred to PEO droplets from now on) with the PEO concentration of $c\lesssim 2\text{ g}/\text{L}$ on specific mesh surfaces. As comparatively illustrated in Fig. 1b,c, ${We}_{C2}$ is about 16.7 and 4.2 for $0.5\text{ g}/\text{L}$ PEO solution on the mesh surface with S = 135 µm and S = 357 µm respectively, while it is 3.6 for $2.0\text{ g}/\text{L}$ PEO solution on the mesh surface with $\text{S}=357\,\upmu\text{m}$, which are close to ${We}_{c1}$ of each mesh surface.

The penetration of recoiling water droplets through mesh pores has been attributed to the impact-induced capillary waves^26,28^. As shown in Fig. 1d,e with a black arrow, the capillary waves are immediately stimulated upon the sudden compression of the water droplet on the mesh surface. They travel along the droplet surface and deform it into a pyramidal structure with several steps that can be observed in a specific range of impact velocities on a solid surface^36^. These water steps gradually merge into one, close to the surface with the ongoing droplet spreading, and a single spire is formed at the droplet center shortly afterward. The subsequent downward motion of the spire creates a cylindrical cavity in the spreading droplet around its maximum extension, as indicated at $2.6\text{ ms}$ in Fig. 1d with a white arrow. Afterward, the droplet retracts, and the cavity shrinks radially inwards. At the same time, a tiny upward jet is emitted from the center of the cavity at specific impact velocities and meshes ${\text S}\lesssim 135\,{ \upmu \text{m}}$ [see 4.6 ms in Fig. 1d]. Following that, the cavity collapse from the center symmetrically due to its biconcave shape that forms a neck at the center of the cavity, squeezing the central jet as indicated at 4.6–4.9 ms in Fig. 1d and the schematic diagram of Fig. 1i. This biconcave shape of the cavity is due to the effect of surface tension that tends to reduce the surface energy of the cavity interfaces. It should be mentioned that the average velocity of the top, center, and bottom of the cavity is approximately the same for the water droplet (see Table S1). This type of cavity collapse is typically observed on superhydrophobic surfaces^18,19^. The cavity walls collide on the central jet, and the flow momentum is redirected from the radial direction to the upward and downward directions. The upward jet tries to elongate and modify the droplet toward the rebounding, whereas the downward jet pushes the liquid toward the superhydrophobic meshes and penetrates the mesh pores. The downward jet is applied on a very small area (${R}_{j}$) with a higher velocity (*v*_*j*_) than the impact velocity (*V*_*0*_) (see Fig. S4), inducing higher pressure on the mesh during the retraction than the pressure generated by water droplet during the impact and spreading at the same Weber number. Therefore, the penetration is observed sooner during the retraction than spreading, *We*_*Cr2*_ < *We*_*Cr1*_ as shown in Fig. 1a. By increasing the impact velocity, the penetration is observed both during the spreading and recoiling (see Fig. 1a,e).

For the symmetric collapse of the air cavity, the characteristic flow velocity and the characteristic length of the flow field in the upward and downward directions should be in the same order. Considering the jet radius ${R}_{j}$, and jet velocity ${v}_{j}$, the kinetic force induced by the downward jet can be predicted as ${\text {F}}_{j}\sim {m}_{j}{v}_{j}^{2}/{R}_{j}$, where the mas of the jet is ${m}_{j}\sim {{\rho R}_{j}}^{2}{\tau }_{j}{v}_{j}$. Characteristic time scale ${\tau }_{j}$ is the inertial-capillary timescale of the jet ${\tau }_{j}\sim ({\rho {R}_{j}^{3}/\gamma )}^{1/2}$ ^19,37^ that can be achieved by equality of the jet inertial in the order of ($\rho {R}_{j}/{\tau }_{j}^{2}$) and the capillary of $\gamma /{R}_{j}^{2}$ ^37^. In this case, the dynamic pressure induced by the downward jet, ${P}_{Dj}$, can be calculated by dividing the induce force over the effective area, which is ${A}_{j}\sim {R}_{j}^{2}$ as follow:

1 $${P}_{Dj}\sim \frac{{\text {F}}_{j}}{{A}_{j }}\sim {\rho }^\frac{3}{2}{R}_{j}^\frac{1}{2}{\gamma }^{-\frac{1}{2}}{v}_{j}^{3}.$$

Ryu et al.^26^ used the film thickness and the impact velocity to characterize the penetration pressure, while we measured the upward jet’s radius and velocity to calculate the jet’s dynamic pressure. The radius and velocity of the upward jet were measured when the jet with a well-defined profile emerged from the impinging droplet at its center. In the image processing, the error of determining droplet profile was typically 1–2 pixels. Given the image resolution of 13 μm/pixel, a standard deviation of 13–26 μm for the jet radius and 0.05–0.10 m/s for the jet velocity have been obtained. The average jet velocity was measured using 10 frames after the well-defined profile appeared.

**Figure 2:**
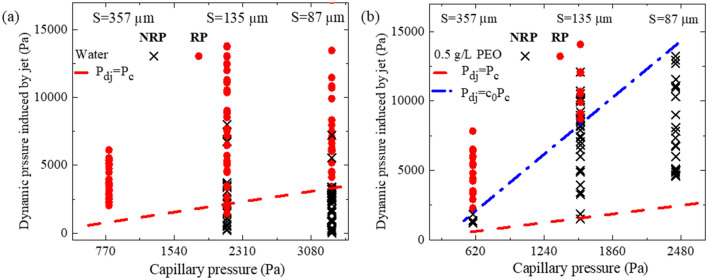
Diagram of the penetration during the reoiling in terms of dynamic pressure induced by jet (${P}_{Dj}\sim {\rho }^{3/2}{R}_{j}^{1/2}{\gamma }^{-1/2}{v}_{j}^{3}$) versus the capillary pressure $4\gamma /S \text{cos}({\theta }_{a})$ for (**a**) Water droplet, (**b**) 0.5 g/L, PEO aqueous droplet. The symbols represent different spray phenomena, the solid symbols show the recoil penetration, and the cross symbol (x), shows no recoil penetration. The dashed line shows ${P}_{Dj}$=${P}_{c}$ and the dashed-dotted line shows ${P}_{Dj}$=${c}_{0}{P}_{c}$ when *c*_*0*_ is the correction coefficient.

Meanwhile, the capillary pressure ${P}_{C}\sim 4\gamma \text{cos}({\theta }_{a}) /\text {S}$ ^26,38^ prohibits the fluid from penetrating the meshes. As a result, the onset of penetration can be predicted by the equality of ${P}_{Dj}$ and ${P}_{C}$ as depicted in Fig. 2a with red dashed line which shows good agreement with experimental data of pure water. For S = 357 μm the recoil penetration is observed as far as the jet forms. Therefore, there is no data point at lower dynamic pressure because the impact velocity was insufficient to form the cavity and the jet.

For the case of the water droplet, the penetration was observed initially during the droplet recoil by increasing the Weber number to ${We}_{c2}$. However, for the PEO droplet, the penetration was initially detected both during the spreading and recoiling (see Fig. 1b,g, Video S5). Upon the impact of the PEO droplet for $We\gtrsim {We}_{c2}\simeq {We}_{c1}$, the droplet penetrates the mesh and forms the ligaments. The penetrated ligaments retract back to the mother droplet before and during the droplet recoiling depending on Weber number [see 2.0–3.0 ms in Fig. 1g]. A slightly deformed cylindrical cavity is formed with the same mechanism of water droplets when the deformation is induced by higher interaction between the surface and PEO mixture^39^, and the upward flow generated by retraction of the ligaments. The upward flow formed around the cavity interface can speed up the retraction of the cavity interface near the mesh surface and changes the cavity dynamics. When in the case of water droplets, the penetrated ligaments detach and form satellite droplets (see Fig. 1f). For PEO droplet, the cavity makes a vase shape during the retraction, as depicted in Fig. 1j and denoted with a green arrow at $4.5\text{ ms}$ in Fig. 1g, due to the faster retraction of the droplet near the mesh surface compared to the top of the droplet (see Fig. S3, Table S1). This faster retraction is formed by the upward flow induced by the retracting ligaments. Eventually, the bottom of the cavity detaches from the mesh surface, moves upward under the effect of the surface tension, and creates an upward jet. During this process, the flow direction is mainly changed to the axial direction to supply the upward jet and induces a downward flow with a lower velocity, which forms the penetration as sketched in Fig. 1j. Therefore, the upward jet's velocity in PEO droplets is higher (see Fig. S4) and the downward flow velocity is lower than water droplets, which explains the higher onset for penetration during the recoiling in PEO solution compared with water. The upper penetration threshold can be limited by the chaotic impact of the retracting interfaces and the upward flow induced by the recoiling of the ligaments which are penetrated in the spreading stage. Unlike the water droplets, which ligaments are destabilized and fragmented, they retract back to the main droplet for the PEO solution that can change the cavity shape and play the role of counterflow to reduce the velocity of the downward flow and suppress the penetration (Video S2). Therefore, the penetration during the recoiling is observed in the narrower the narrower range of Weber number compared with water (see Fig. 1a–c). As it is presented in Fig. 2b the upward jet characteristics no longer represent the downward flow where the pressure measured for the onset of penetration is nearly *c*_*0*_ = 5 times higher than capillary pressure (see the blue dashed-dotted line in Fig. 2b).

By increasing the solid fraction (reducing the hole size), and PEO concentration, the recoil penetration is removed, as shown in Fig. 1b,c, which can be rationalized by a more significant interaction of high concentration PEO solution with a larger surface area and increasing the extensional viscosity^40^ that reduces the upward jet velocity (Fig. S4) and the subsequent downward flow velocity, inhibiting penetration during the recoiling.

### Ligaments dynamics

For $We\gg {We}_{c1}$, the liquid penetrates the mesh and creates long and smooth ligaments. A spike droplet is generated at the top of the ligaments. The formation of the spike is stimulated by the well-known Rayleigh-Plateau instability. The instability originated from the impact-induced perturbation and the wire mesh vibration^25,31^. These perturbations deform the interface and reduce the surface area, which is favored by surface tension^41^. Thereafter, the surface tension tries to minimize the surface area and form the elliptical shape of the spike.

**Figure 3:**
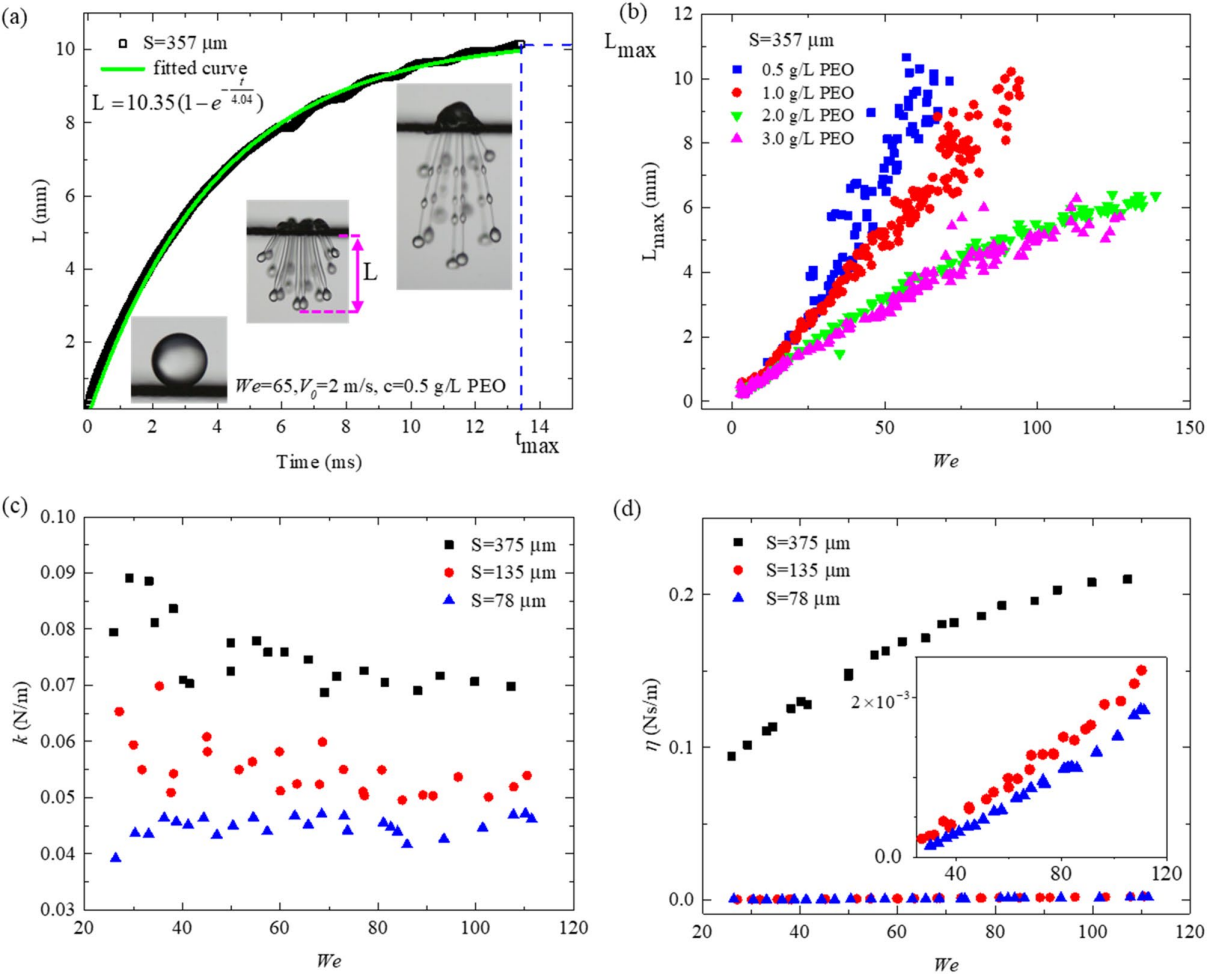
The ligament dynamics. (**a**) Tiem evolution of the ligament length for meshes with pore sizes S = 357 μm at *We* = 66 and 0.5 g/L PEO concentration. The solid green line shows the fitted curve by the spring-damper equation of motion. (**b**) The maximum ligament length versus the Weber number for meshes with pore sizes S = 357 μm and different PEO concentrations. (**c**) The spring constant and the (**d**) damper coefficient were extracted from the spring-dashpot modeling.

Surprisingly, these ligaments retract to the mother droplet in a wide range of Weber numbers^31^ (see Fig. 1h). The reason is hidden in the contest between the elastic force and the surface tension when the elastic force is powerful enough to tackle the surface tension and the gravity force trying to collapse the ligament. Increasing the Weber number, the ligaments are destabilized due to the Rayleigh- Plateau instability^25^, and long waives perturbations and small elliptical-shaped (crests swell^25^) droplets are created on the ligaments at $c=0.5 \, \text{and} \, 1 \text{g/L}$ (see the inset of Fig. 3a, Video S6). These crest swell drops move down on the ligaments due to gravity and merge with the spike or each other. Crest swell drops finally detach at the higher Weber number when *c* = 0.5 g/L. For higher PEO concentrations $c>1 \text{g/L}$, the crest swell drops do not form, and the ligaments stop at the maximum length, retracting back fully to the mother droplet. Historical evolution of the central ligament’s size is presented in Fig. 3a till the maximum length (L_max_).

The maximum length of the ligaments (L_max_) before retraction was measured and presented in Fig. 3b. Increasing the PEO concentration boosts the elasticity of the mixture where the molecular chain acts like a stronger hook spring^42^. As a result, the penetrated ligaments are shorter at the same impact velocity. Moreover, the ligament length increases by Weber number due to increasing the momentum of the fluid when its thickness decreases. Indeed after a certain Weber number, the mass of the liquid that penetrates the mesh reaches a constant value, as presented by Soto et al.^23^. Therefore, at higher Weber numbers, the same volume of liquid is injected into the mesh pores with higher momentum creating thinner and longer ligaments.

The ligaments evolution is modeled by a system of parallel spring and dashpot with a spring constant of (*k*) and damper coefficient of (*η*), which is stretched by a constant impact force $\text{F}=\uprho {{V}_{0}}^{2}{\text{S}}^{2}$ as presented schematically in Fig. S5c. Using the force balance, we can write $\text{F}-{k \text{L}}-\upeta \dot{\text{L}}=0.$ Where L is the length of ligaments at time t. The empirical modeling shows that the ligament growth indeed follows the solution of the spring-dashpot equation with boundary condition L(0) = 0, as shown in Fig. 3a.

2 $$\text {L(t)}=\left(\frac{F}{k}\right)\left(1-{e}^{-\frac{kt}{\eta }}\right).$$

The value of the (F/*k*) can be defined as the maximum length of the ligament L_max,_ and the modulus ratio can be defined as $\zeta =\eta /{k},$ which can be extracted from the fitting parameters of ligament size evolution (Fig. 3a). Equation (2) can be reformed as follow:

3 $$\text{L}\left(\text{t}\right)={\text{L}}_{\text{max}}\left(1-{\text{e}}^{-\frac{t}{\zeta }}\right).$$

The spring constant *k* = F/L_max_ and consequently the damper coefficients $\eta =\zeta /{k}$ can be calculated by defining the force induced on the fluid. Hu et al.^43^ have monitored the impact force of the droplet on a superhydrophobic surface and reported that the peak force is around the (0.87–87) times of induced dynamic pressure force $\rho {V}_{0}^{2}{D}_{0}^{2}$. Therefore, we used the dynamic pressure to determine the force as $\text{F}=\rho {{V}_{0}}^{2}{\text{S}}^{2}$, where S^2^ is the pore area that the pressure applied on it. The results are presented in Fig. 3c,d. The spring constant, which is almost independent of the impact velocity (see Fig. 3c) represents the elastic force F_e_ induced by polymer additives and the surface tension force induced by the connection of the spike to the ligament$,$ which is against the ligament growth direction^44^ and counted as a resistance force. Assuming a uniform ligament thickness $\delta$, the surface tension force can be calculated as ${\text{F}}_{\gamma }= \pi \delta \gamma$ in the order of ${10}^{-5}$ N. The extracted data shows that F_e_ is in the order of magnitude ${10}^{-3} \text{to}{ 10}^{-6}$ N when the elastic force increases by impact velocity and the pore size as more polymer molecules are injected at larger pore sizes, and polymers stretch more at higher velocities (see Fig. S6). The dashpot constant denotes the viscous dissipation, which increases with impact velocity and pore sizes (Fig. 3d).

**Figure 4:**
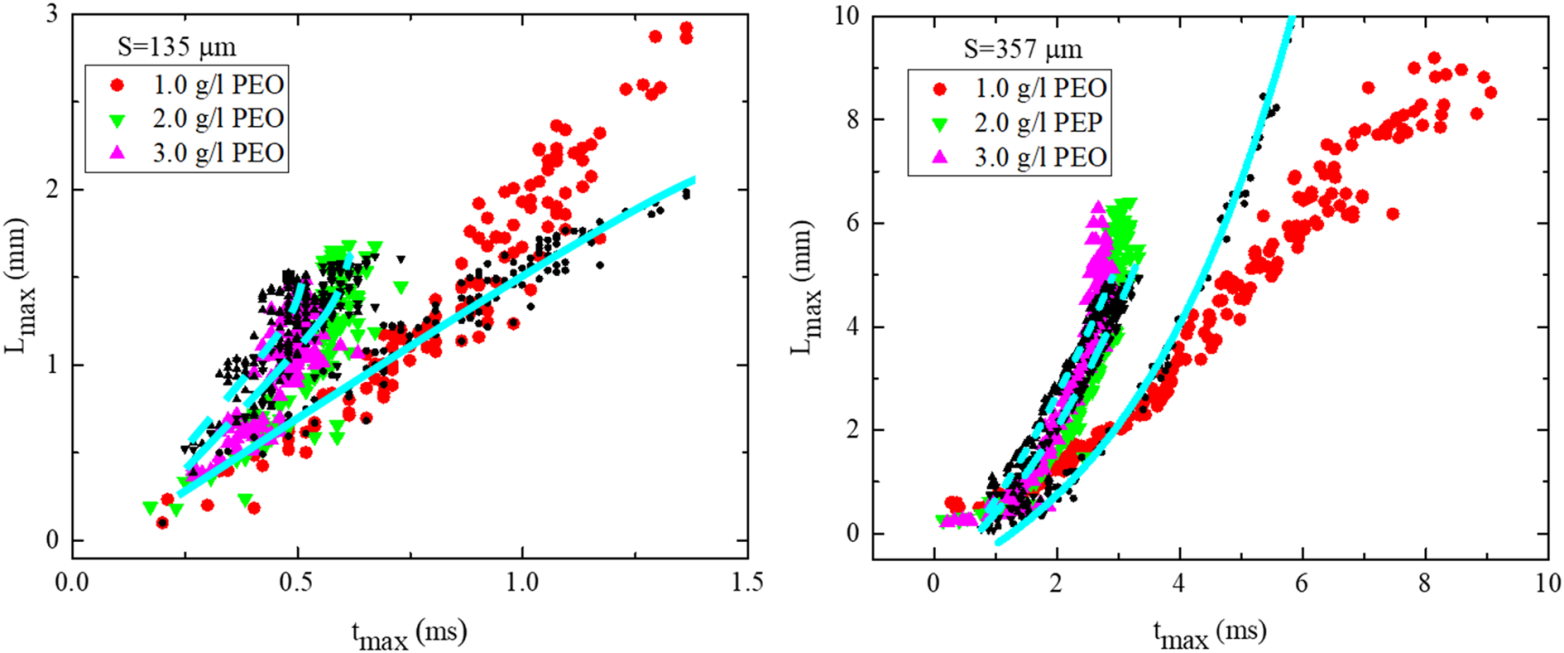
The maximum ligament size versus its corresponding time as defined in Fig. 3a. The black symbol shows the modeling result using ${\text{L}}_{\text{max}}=({\Lambda }_{p}-1/6\pi {d}^{3})/\frac{\pi }{4}{\delta }^{2}+d$, the cyan lines depicted the modeling trend. In the left figure S= 135 μm and in the right figure S=357 μm.

Besides, the maximum length of the ligaments can be modeled by the conservation of the mass. The ligament can be modeled as a long uniform cylinder of fluid with a spherical spike at the top of the ligament. By measuring the average spike diameter (*d*) (Table S2) and approximating the thickness of the ligament as $\delta \sim \text{exp}(-t/2\lambda$) or $\delta \sim \text{exp}(-t/3\lambda$)^45,46^ with experimental fitting (see Fig. S7) and the penetrated volume ${\Lambda }_{p}={1.5\Lambda }_{0}{(\text {S}/{D}_{0})}^{2}(1-\frac{{V}_{cr1}}{{V}_{0}})$ ^23^, the maximum ligament size can be calculated as ${\text{L}}_{\text{max}}=({\Lambda }_{p}-1/6\pi {d}^{3})/\frac{\pi }{4}{\delta }^{2}+d$. Where Λp is the volume of the liquid that penetrated into a single pore at the impact point and Λ0 is the initial volume of the droplet. Although the modeling is so simple and does not include the complex details of the ligaments dynamics but still can capture the whole picture of the ligament evolution as presented in Fig. 4 and Fig. S8.

## Conclusion

In conclusion, we demonstrated that the penetration during the spreading of viscoelastic droplet happens with the same threshold of water droplet when adding a trivial amount of PEO to the water droplet can effectively stabilize the ligaments and suppress the spraying. The penetrated ligaments grow, reach the maximum size, and retract back to the mother droplet. The ligaments’ evolution until the maximum size was modelled by the parallel spring-dashpot Kelvin-Voigt model of viscoelasticity. The results show that for the PEO aqueous droplets, penetration during the droplet recoiling happens with a mechanism distinct from water droplets. The PEO additive also affects the lower and upper penetration threshold during the droplet recoiling by changing the cavity dynamics and the upward flow induced by recoiling ligaments.

## Experimental method

Superhydrophobic meshes with different geometries (pore size S = 87 μm, 135 μm, and 357 μm) were fabricated by first growing hairy Cu(OH)2 nanostructures (Fig. S1) on smooth copper meshes in the aqueous solution of NaOH/ammonium persulfate and then coating them a thin layer of polydimethylsiloxane (monomer/cross-linker ratio of 10:1, Sylgard 184) ^31^. Impact experiments were performed using pure water and aqueous solutions of polyethylene oxide (PEO) with a molecular weight of $4\times {10}^{6}$ and four mass concentrations *c* = 0.5 g/L, 1.0 g/L, 2.0 g/L and 3.0 g/L. Droplets with diameter ${D}_{0}\approx 2.0$ mm were released from a blunt needle and vertically fell onto the superhydrophobic meshes placed underneath at a velocity of *V*_*0*_ = 0.1–2.8 m/s. The dynamic behaviors of the impinging droplets were captured using a high-speed Phantom camera at 52,000 fps. The rheological properties of the aqueous solution were measured using a standard rheometer (Physica MCR 301, Anton Paar), and presented in previously published studies ^47^.

## Supplementary Information

Supplementary Information.

Supplementary Video 1.

Supplementary Video 2.

Supplementary Video 3.

Supplementary Video 4.

Supplementary Video 5.

Supplementary Video 6.

## Data Availability

The datasets generated during and/or analyzed during the current study are available from the corresponding author on reasonable request.
