# Supplementary material for: Penetration and ligament formation of viscoelastic droplets impacting on the superhydrophobic mesh
Source: Sci Rep. 2022 Jul 13;12:11920. doi: 10.1038/s41598-022-15645-1 (PMC9278331; doi:10.1038/s41598-022-15645-1)
Supplement: Supplementary file 1 — Supplementary Information. [file 41598_2022_15645_MOESM1_ESM.pdf]

Penetration and ligament formation of viscoelastic droplets impacting on the superhydrophobic mesh

Abbasali Abouei Mehrizi[1*], Shiji Lin[1], Lijie Sun[1], Yile Wang[1], Longquan Chen[1*]

[1]*School of Physics, University of Electronic Science and Technology of China, Chengdu 610054, China.*

* *Authors to whom correspondence should be addressed, abbasabouei@gmail.com, lqchen@uestc.edu.cn.*

The supplementary materials cover the necessary figures and explanation that are cited in the original manuscript. The supplementary materials are included a text file and six videos. The text includes the details of the mesh surface used in the current study. The critical impact velocities for penetration through the mesh are presented in a separate figure. The cavity retraction and its velocity are monitored to detect the penetration mechanism. The upward jet velocities are compared for water and PEO droplets. The details about the evolution of the ligaments and their modeling are presented. The videos contain water and PEO-Water mixture droplets impact on the superhydrophobic mesh at different impact velocities when distinct phenomena happen.

The video titles are as fellow:
Video S1- Water penetration during the spreading.
Video S2- PEO penetration during the spreading.
Video S3- Water penetration during the recoiling.
Video S4- Water, Penetration during the spreading and recoiling.
Video S5-PEO, Penetration during the spreading and recoiling.
Video S6- PEO, ligament formation.

The figures are as follow:
FIG. S1. SEM image of the copper mesh and the Nano-structures on its surface after superhydrophobic treatment in addition to the physical characteristics of the meshes.
FIG. S2. Variation of the critical impact velocity for the penetration during the spreading for different PEO concentrations and pore sizes.
FIG. S3. Comparison of the cavity diameter evolution at the top, middle, and bottom of the cavity as a function of time (a) water droplet, (b) PEO droplet, *c*=1 g/L. The impact velocity is $V_0 = 0.6\ \mathrm{m/s}$ and $\mathrm{S} = 135\ \mu\mathrm{m}$. The cavity forms at t=0.
FIG. S4. Variation of upward jet velocity formed after collapsing/detachment of the air cavity during the retraction versus the impact velocity for water/different PEO concentrations, the mesh pore size is 135 μm. Penetration during the droplet recoil is observed for water droplets with impact velocities in the range of $0.7<V_0<1.67$m/s and for 0.5 g/L PEO mixture in the range of $0.76<V_0<1$ m/s. The recoil penetration is not observed for 1 g/L and 2 g/L PEO concentrations on mesh with S=135 μm due to the higher interaction between the liquid and substrate.
FIG. S5. Time evolution of the ligament length for meshes with (a) different pore sizes at *We*=66 and 0.5 g/L PEO concentration, and (b) different PEO concentrations at *We*=66, an S=375 μm. The

solid green line shows the fitted curve by the spring-damper equation of motion. The fitting parameters are L_{max}=10.35 and $\lambda = 4.04$, the coefficient of determination $R^2 = 0.997$. (c) Schematic diagram of the spring-damper system was used to model the evolution of the ligaments.
Fig. S6. Variation of the estimated elastic force by We number for different mesh sizes and c=1 g/L PEO concentration.
FIG. S7. Evolution of the ligament thickness versus time at different PEO concentrations and impact velocities of mesh with S=357 μm.
FIG. S8. Comparison of the experimental maximum ligament sizes for meshes with (a) S=357 μm and (b) S=135 μm for different PEO concentrations. The black data points are the modeling results, and the cyan lines show their trend. The left graphs are zoomed in and presented on the right side.

The tables are as follow:
Table S1. The average velocity of the interface of the cavity during the retraction at the top, middle, and bottom of the cavity for water and different PEO droplets at $V_0 = 0.6$ m/s.
Table S2. The average value of the spikes diameters. The spikes are not spherical but elliptical, so the average diameter of the spikes is calculated using the geometric mean of the ellipse's maximum and minimum diameter sizes.

Viscoelastic droplet impact on superhydrophobic wire meshes was investigated. Fig. S1 shows the SEM Image and physical parameters of the mesh surfaces used in the current study.

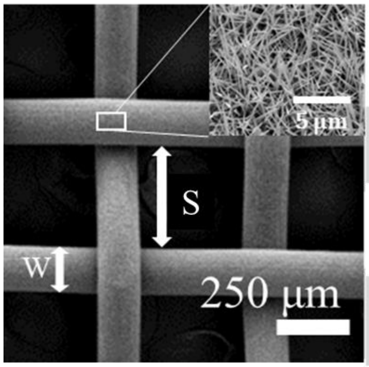

W(μm)	S (μm)
79	87
114	135
161	357

FIG. S1. SEM image of the copper mesh and the Nano-structures on its surface after superhydrophobic treatment in addition to the physical characteristics of the meshes.

Penetration during the spreading.

Upon the impact, the droplet penetrates the mesh after a threshold velocity. The critical We number is calculated based on the equality of dynamic pressure and the capillary pressure as $We_{cr1} = \frac{4D_0}{S}\cos(\theta_a)$ which shows that We_{cr1} is independent of the fluid characteristics. Fig. S2 shows that the PEO concentration does not considerably affect the critical weber number as expected, although a small increment is observed.

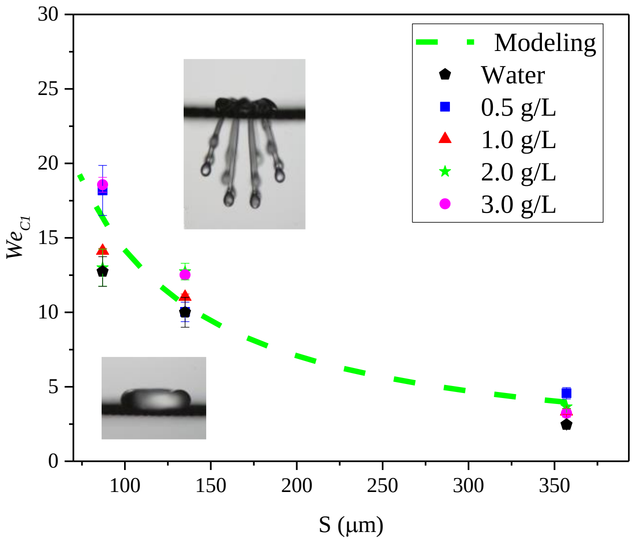

FIG. S2. Variation of the critical impact velocity for the penetration during the spreading for different PEO concentrations and pore sizes.

***Penetration during the* recoiling**

The penetration during the recoiling of the water droplet is observed due to the formation and subsequent collapse of the air cavity. The evolution of the cavity diameter was monitored at three local positions, namely the top, middle, and bottom of the cavity. Fig S3. (a) and Table S1 show that the top, middle, and bottom of the cavity retract with approximately the same velocity for water droplets, keeping the cavity shape cylindrical (biconcaved shape) in the whole retraction procedure till the collapse time. The retracting interfaces impact each other after the cavity collapse, which induces upward and downward jets, leading to liquid penetration into the meshes. The upward jet velocity and size can be used as characteristic parameters to calculate the pressure induced by the downward jet on the mesh (penetration pressure) when the air cavity collapse is symmetrical for water droplet impact.

However, in the PEO droplet cases, the cavity's bottom retracts faster than the top, which forms a vase shape cavity (see Fig. S3 (b) and Table S1). The vase-shaped cavity eventually detaches, moves upward, and induces an upward jet and a downward flow that forms the penetration. Due to the difference in the mechanism of the formation of the upward jet, the upward jet velocity in the PEO droplets is higher than in the water droplets, as shown in Fig. S4.

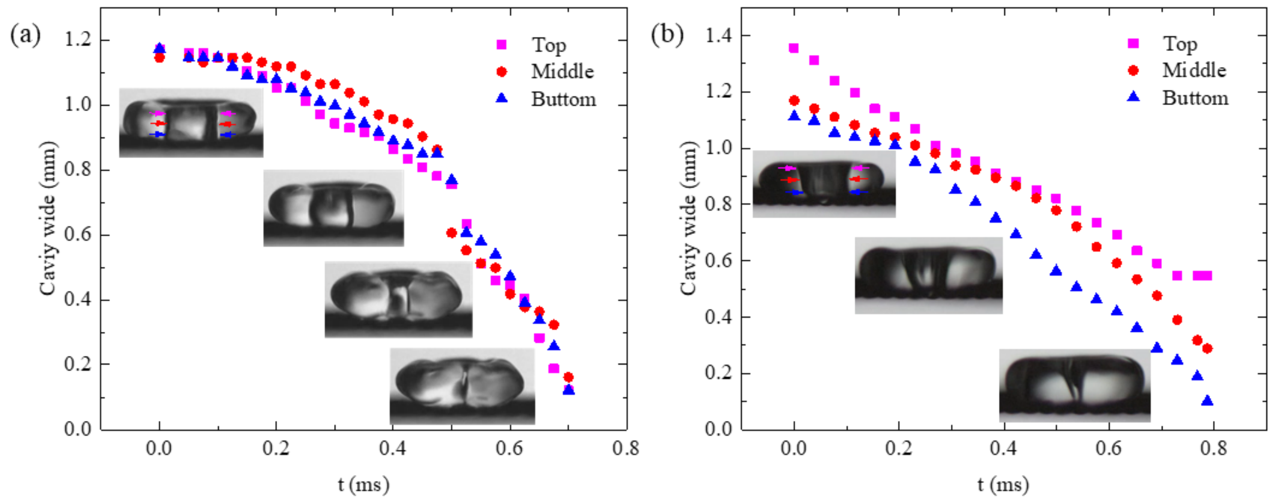

FIG. S3. Comparison of the cavity diameter evolution at the top, middle, and bottom of the cavity as a function of time (a) water droplet, (b) PEO droplet, *c*=1 g/L. The impact velocity is $V_0 = 0.6\ \mathrm{m/s}$ and $\mathrm{S} = 135\ \mu\mathrm{m}$. The cavity forms at t=0.

Table S1. The average velocity of the interface of the cavity during the retraction at the top, middle, and bottom of the cavity for water and different PEO droplets at $V_0 = 0.6\ \mathrm{m/s}$.

	Average velocity (m/s)		
	Top	Middle	Bottom
Water	1.50	1.51	1.50
0.5 g/L PEO	1.17	1.24	1.47
1.0 g/L PEO	1.03	1.12	1.28
2.0 g/L PEO	1.12	1.22	1.22
3.0 g/L PEO	1.16	1.12	1.25

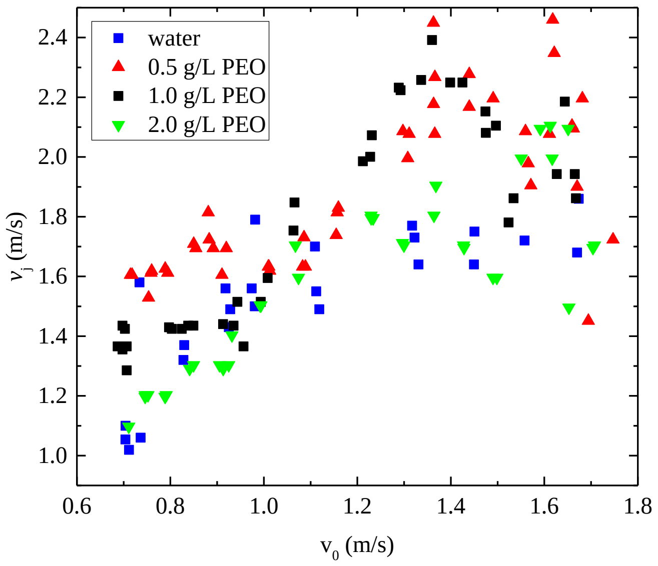

FIG. S4. Variation of upward jet velocity formed after collapsing/detachment of the air cavity during the retraction versus the impact velocity for water/different PEO concentrations, the mesh pore size is 135 µm. Penetration during the droplet recoil is observed for water droplets with impact velocities in the range of $0.7<V_0<1.67$m/s and for 0.5 g/L PEO mixture in the range of $0.76<V_0<1$ m/s. The recoil penetration is not observed for 1 g/L and 2 g/L PEO concentrations on mesh with S=135 µm due to the higher interaction between the liquid and substrate.

Ligaments dynamics

The growth of the ligaments can be modeled by the parallel spring-damper viscoelastic model. Upon the impact, the liquid penetrates, and the ligaments grow linearly with time till the droplet completely spreads over the surface and reaches the maximum diameter. At this point, there would be no penetration of the liquid into the mesh pores. Afterward, the penetrated ligaments start to stretch, increasing the ligaments' length and decreasing the ligaments' wide till reaching the maximum length as shown in Figs. S5(a) & S5(b). Neglecting the gravity, we simply assumed that the growth of the ligaments is only due to the impact force, and it is constant during the whole ligament growth. Therefore we model the evolution of ligaments as a parallel spring-damper system when a constant force is imposed on them with a very low damping coefficient (see Fig. S5 (c)). The spring constant represents the elastic properties of the liquid (F_e) and the surface tension force induced by the connection of the spike to the ligament, which is against the ligament growth direction[1] (F_γ), and the damper constant represents the effect of liquid viscosity. Figure S5(c) shows the schematic diagram of the spring-damper model. The well-known solution for the spring-damper differential equation, $\mathrm{F} - k\mathrm{L} - \eta\dot{\mathrm{L}} = 0$, with

boundary condition L=0 at *t*=0 leads to the following equation.

$$L(t) = (\frac{F}{k})(1 - e^{-\frac{kt}{\eta}}) \quad (S1)$$

The value of the (F/k) can be defined as the maximum length of the ligament L_{max}, and the modulus ratio can be defined as $\zeta = \eta/k$. The equation S1 can be reformed as follow

$$L(t) = L_{max}(1 - e^{-\frac{t}{\zeta}}) \quad (S2)$$

The spring constant $k=F/L_{max}$ and the damper coefficients $\eta = \zeta/k$ can be calculated by defining the force induced on the fluid. We used the dynamic pressure and defined the force as $F = \rho {V_0}^2 S^2$, where S^2 is the pore area that the pressure applied on it.

Figure S5 (a) and (b) show the time evolution of the ligament length for different pore sizes and PEO concentrations. The fitting green line shows good agreement between the experimental data and the modeling. Figure 3 in the manuscript represents an increasing trend of the spring constant with the pore size. The thickness of the penetrated ligaments is larger for bigger pores; therefore, it is expected to have a larger spring constant. On the other hand, we have a larger ligament length for bigger pores because of the more significant force imposed on the ligaments due to the higher area of applied force, $F = \rho {V_0}^2 S^2$. The extracted data shows that F_{γ} is in the order of 10^{-5} N and F_e is in the order of magnitude $10^{-3}\ to 10^{-6}$ N when the elastic force increases by impact velocity and the pore size, as more polymer molecules are injected at larger pore sizes, and polymers stretch more at higher velocities (see Fig. S6).

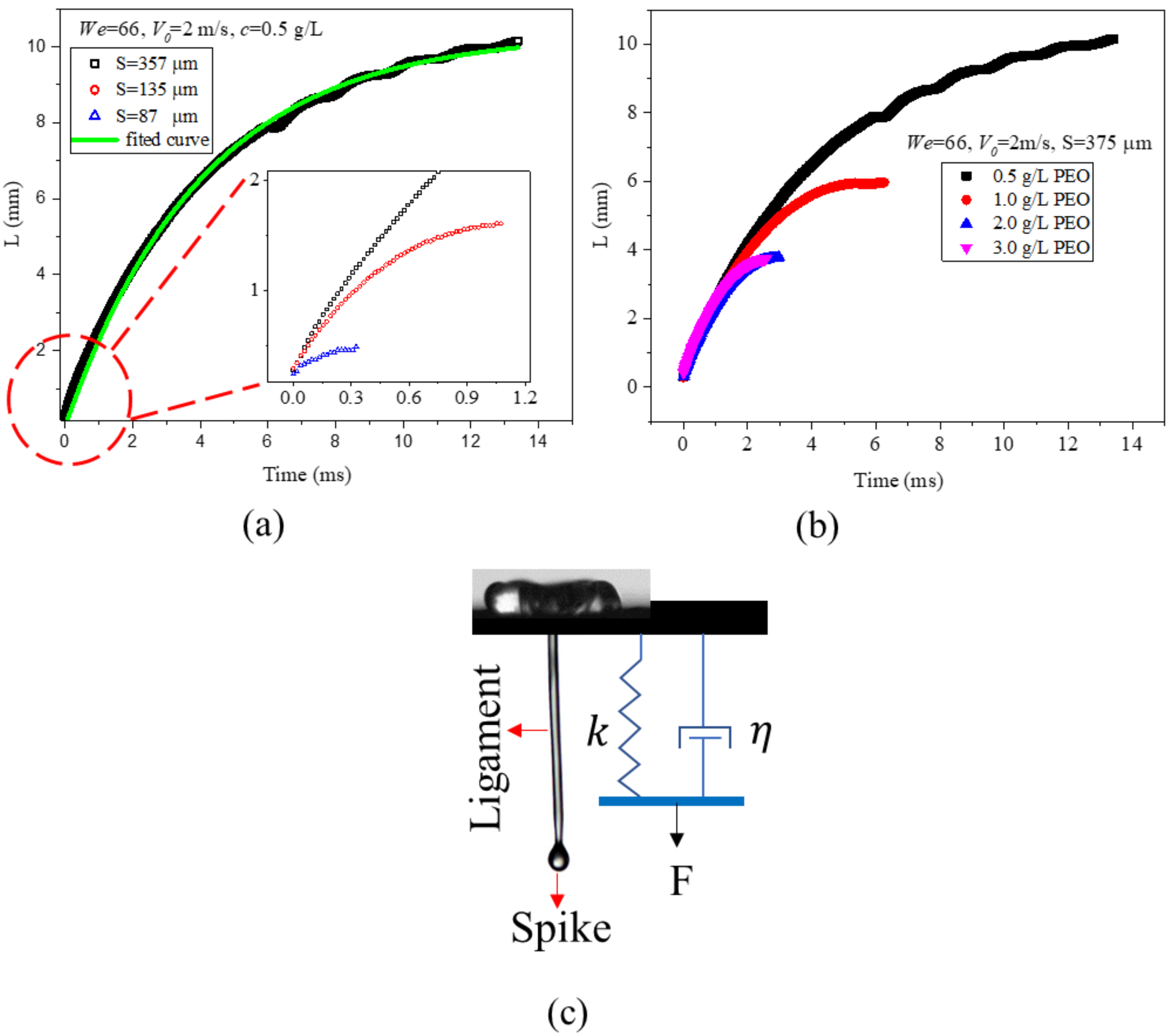

FIG. S5. Time evolution of the ligament length for meshes with (a) different pore sizes at *We*=66 and 0.5 g/L PEO concentration, and (b) different PEO concentrations at *We*=66, an S=375 µm. The solid green line shows the fitted curve by the spring-damper equation of motion. The fitting parameters are L_{max}=10.35 and $\lambda = 4.04$, the coefficient of determination $R^2 = 0.997$. (c) Schematic diagram of the spring-damper system was used to model the evolution of the ligaments.

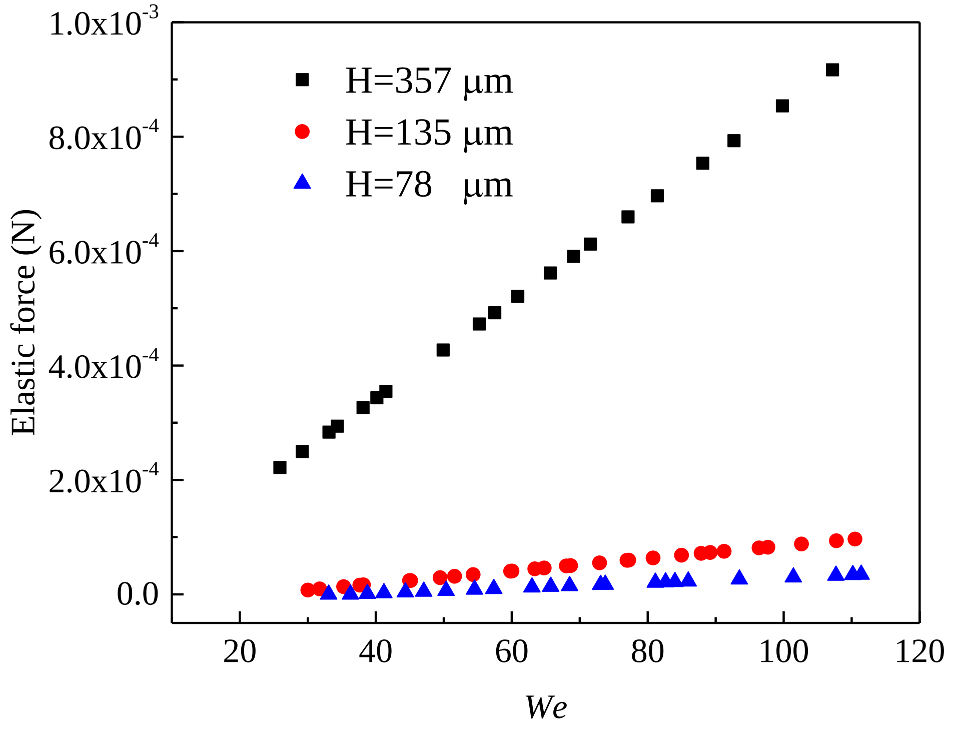

Fig. S6. Variation of the estimated elastic force by We number for different mesh sizes and c=1 g/L PEO concentration.

According to the exponential thinning law, the thinning of the viscoelastic jet can be estimated by $\delta \sim \exp(-t/2\lambda)$ [2] or $\delta \sim \exp(-t/3\lambda)$ [3] when δ is the thickness of the ligament, t is the time, and λ is the relaxation time. We have measured the evolution of ligament thickness for mesh wtih S=375 μm; different PEO concentrations and fitted them with exponential decay equations as presented in Fig. S7. Increasing the PEO concentration slightly affects the thickness variation. The average fitting of all data can be expressed as $\delta = 345\, e^{-t/4.6}$. Therefore, the relaxation time value can be estimated as 2.3 ms or 1.53 ms, which has the same range compared with previous studies [2,4]. Measuring the thickness variation of ligaments is impossible for the mesh with S= 135 μm due to their small length and merging phenomena; therefore, we used the same trend as $\delta = 140\, e^{-t/4.6}$ since the relaxation time is the intrinsic parameter of the liquid.

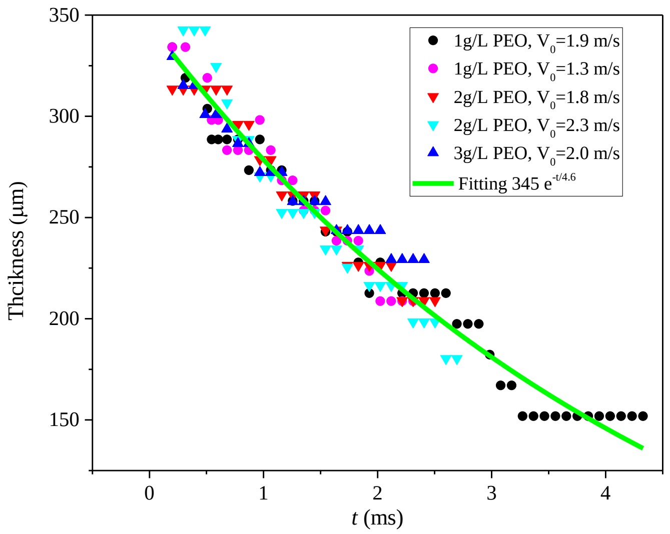

FIG. S7. Evolution of the ligament thickness versus time at different PEO concentrations and impact velocities of mesh with S=357 μm.

The maximum ligament size can be predicted using a simple mass conservation analysis. The ligament at the maximum length is modeled with a uniform cylinder with an attached spherical spike at the end of it. It is assumed that the volume of the penetrated ligament and spike are constant, and the mass does not go back to the mother droplet. By measuring the average diameter of the spikes as presented in table S2, the maximum ligament sizes were easily calculated and presented in Fig. S8 for S=357 μm and S=135 μm and PEO concentrations c=1, 2, and 3 g/L. Although we neglect many details in the modeling, including the non-uniform thickness of the ligaments and small variation in the spike size and the retracted volume to the mother droplet, the modeling can acceptably predict the maximum ligament size and its trend. In the case of 0.5 g/L at a wide range of impact velocities, the ligament destabilized, and several elliptical-shaped drops (crest swell drops) appeared, which moved on the ligaments and merged with the spike at the maximum size of ligaments. Therefore, our simple modeling cannot predict the maximum ligament size.

Table S2. The average value of the spikes diameters. The spikes are not spherical but elliptical, so the average diameter of the spikes is calculated using the geometric mean of the ellipse's maximum and minimum diameter sizes.

	Average spike diameter (mm)		
Mesh pore	1 g/L PEO	2 g/L PEO	3 g/L PEO
S=357 μm.	0.57	0.5	0.45
S= 135 μm.	0.23	0.2	0.19

(a) S=357 µm

(b) S=135 µm

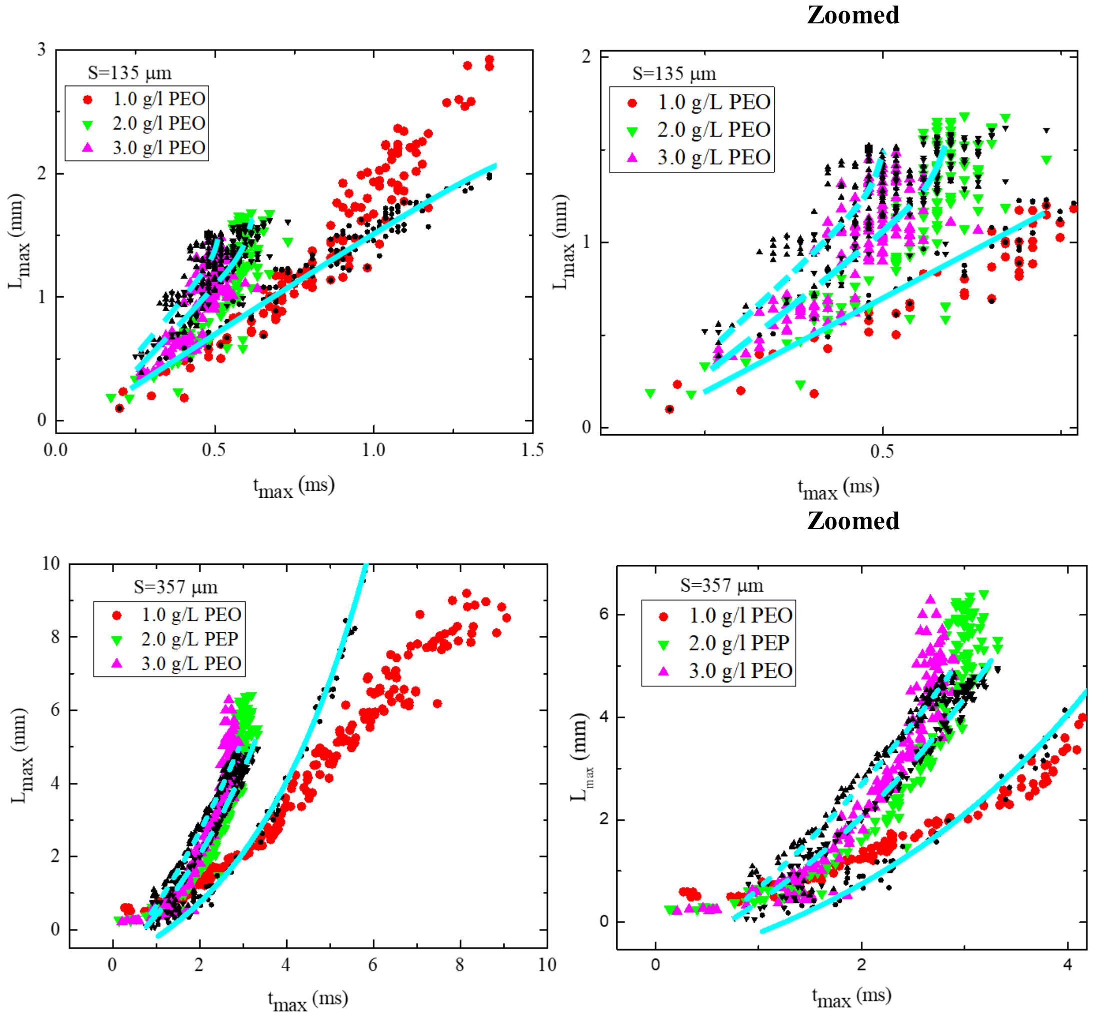

FIG. S8. Comparison of the experimental maximum ligament sizes for meshes with (a) S=357 µm and (b) S=135 µm for different PEO concentrations. The black data points are the modeling results, and the cyan lines show their trend. The left graphs are zoomed in and presented on the right side.

References

1 Smolka, L. B. & Belmonte, A. Drop pinch-off and filament dynamics of wormlike micellar fluids. *Journal of non-newtonian fluid mechanics* **115**, 1-25 (2003).

2 Mathues, W., Formenti, S., McIlroy, C., Harlen, O. G. & Clasen, C. CaBER vs ROJER—Different time scales for the thinning of a weakly elastic jet. *Journal of Rheology* **62**, 1135-1153 (2018).

3 Wagner, C., Amarouchene, Y., Bonn, D. & Eggers, J. Droplet detachment and satellite bead formation in viscoelastic fluids. *Phys. Rev. Lett.* **95**, 164504 (2005).

4 Sur, S. & Rothstein, J. Drop breakup dynamics of dilute polymer solutions: Effect of molecular weight, concentration, and viscosity. *Journal of Rheology* **62**, 1245-1259 (2018).
